# Efficacy and Safety of Immune Checkpoint Inhibitors for Advanced Malignant Melanoma: A Meta-Analysis on Monotherapy Vs Combination Therapy

**DOI:** 10.7150/jca.72210

**Published:** 2022-08-08

**Authors:** Jnaneshwari Pradeep, Thin Thin Win, Saint Nway Aye, Chandrashekhar T Sreeramareddy

**Affiliations:** 1 School of Medicine, International Medical University, 126, Jalan Jalil Perkasa 19, Bukit Jalil, 57000 Kuala Lumpur, Malaysia.; 2 Pathology Department, School of Medicine, International Medical University, 126, Jalan Jalil Perkasa 19, Bukit Jalil, 57000 Kuala Lumpur, Malaysia.; 3 Community Medicine Department, School of Medicine, International Medical University, 126, Jalan Jalil Perkasa 19, Bukit Jalil, 57000 Kuala Lumpur, Malaysia.

**Keywords:** Immune checkpoint inhibitors, advanced malignant melanoma, systematic review, meta-analysis, monotherapy, combination therapy.

## Abstract

**Background:** Immune checkpoint inhibitors (ICIs) are approved as cancer immunotherapeutic agents for advanced malignant melanoma (MM) in recent years, and nivolumab and ipilimumab are the most widely used ICIs either alone or in combination. However, their efficacy and safety between single and combined ICIs are not clear. This meta-analysis (MA) is aimed to update the efficacy and safety of ICIs by comparing monotherapy and combination therapy in the treatment of advanced MM.

**Method:** We searched PubMed, Embase, EbscoHost and ClinicalTrials.gov for the eligible randomized controlled trials (RCTs) which compared the efficacy and safety of ICIs between a single ICI and combined ICIs. The outcomes analyzed included overall survival (OS), progression-free survival (PFS), objective response rate (ORR) and treatment-related adverse events (AEs). A fixed-effect or random-effects model was adopted depending on the study heterogeneity.

**Results:** A total of nine RCTs were included in this MA. Regarding the efficacy, combined nivolumab and ipilimumab therapy showed statistically significant prolonged OS and PFS with HR 0.65, 95% CI [0.53, 0.79], p <0.0001 and HR 0.48, 95% CI [0.38, 0.60], p<0.0001 respectively. Combination therapy with nivolumab and ipilimumab also showed statistically significant longer ORR than monotherapy; with RR 2.15, 95% CI [1.63, 2.84], p <0.00001. In terms of safety, the incidence of all AEs which include any AEs, high-grade, haematological, gastrointestinal, dermatological, pulmonary, liver and endocrine AEs were significantly lower with monotherapy (either nivolumab or ipilimumab) of ICI compared to combination ICI therapy with a p-value <0.00001 to 0.03.

**Conclusion:** Efficacy of the combined nivolumab and ipilimumab was better than a single ICI, especially in the treatment of advanced MM. Although combination therapy showed better efficacy than monotherapy, monotherapy (either nivolumab or ipilimumab) was safer than combination therapy as it tended to decrease the incidence of most of the treatment-related AEs.

## Introduction

Malignant melanoma (MM) is a malignant neoplasm arising from melanocytes, the melanin-producing cells of the body. It is one of the serious skin cancers and is the fifth and sixth most common cancer in males and females respectively in the United States 1. Stage I and II MM are localized, considered early stage and curable by complete resection with a 5-year survival rate of 99.4%. However, the prognosis of regional and distant metastatic MM (stages III and IV, respectively) is generally poor, with a 5-year survival rates of 60% in stage III and 16% in stage IV 2. The poor prognosis of advanced MM is partly due to the limited available therapeutic options 3.

In recent years, immunotherapy with immune checkpoint inhibitors (ICIs) has improved the treatment of different types of cancer including MM. Immunological checkpoint molecules suppress the attack of tumour-specific T cells, which are an integral part of anti-tumour immunity. Some ICIs block the interaction between programmed cell death-1 (PD-1) on T cells and its ligand PD-L1 on cancer cells and myeloid cells 4-6. Other ICIs target cytotoxic T-lymphocyte antigen-4 (CTLA-4) which blocks negative signals during T-cell interaction with antigen-presenting cells and depletes regulatory T cells, thereby restoring and enhancing T-cell reactivity 7.

Novel immuno-oncologic therapies such as anti-CTLA-4 antibodies (ipilimumab) and anti-PD-1 antibodies (nivolumab and pembrolizumab) have recently achieved remarkable outcomes against advanced MM in clinical trials, with significant survival benefits and manageable safety outcomes 8-12. ICIs can improve overall survival in both sexes with some types of advanced cancers such as MM, non-small cell lung cancer and renal cell carcinoma 13.

Nivolumab and pembrolizumab of PD-1 inhibitors, and ipilimumab of CTLA-4 inhibitors are approved by FDA in 2014 as cancer immunotherapeutic agents 14, 15; and nivolumab and ipilimumab are the most widely used ICIs in the treatment of advanced MM, either alone or in combination 16. The systematic review (SR) on ICIs comparing monotherapy and combination therapy on advanced solid cancers which was published in 2021 included only three randomized control studies (RCTs) for advanced MM 17. The meta-analysis (MA) on efficacy and safety between monotherapy and combination ICIs in advanced MM was published in 2017 18. To fill this literature gap after the updated literature search, we aimed to perform this MA to update the efficacy and safety of ICIs comparing monotherapy and combination therapy; and to provide up-to-date comprehensive evidence for clinical decision making to choose different treatment options available in the treatment of advanced MM.

## Methods

The SR and MA were performed in accordance with the updated guideline of Preferred Reporting Items for Systematic Review and Meta-Analyses (PRISMA) statement 19.

### Identification of eligible studies

The systematic literature search was carried out in health-related electronic databases such as PubMed, Embase, EbscoHost and Cochrane library. Clinical trial studies were also searched at ClinicalTrials.gov. The search terms were immune checkpoint inhibitors, immunotherapy, ipilimumab, nivolumab, pembrolizumab and advanced malignant melanoma. The search was started in March 2021, and it was limited to the original articles published in the English language up to July 2021. To find out additional studies, reference lists of the original articles were also screened.

### Inclusion and exclusion criteria

Studies in the selected articles were to meet five criteria of PICOS format: (1) Participants: individuals with histopathologically diagnosed advanced MM (Stage III and IV) of any age and sex; (2) Intervention: combined ICIs including nivolumab or pembrolizumab and ipilimumab simultaneously regardless of the dose; (3) Comparison: nivolumab or pembrolizumab or ipilimumab alone; (4) Outcomes: primary outcomes were overall survival (OS) which is defined as the time from randomization to death from any cause and treatment-related adverse effects (AEs). The secondary outcomes included progression free survival (PFS) which is defined as the time from randomization to cancer progression or death from any cause and objective response rate (ORR). (5) Studies: parallel RCTs reporting on the efficacy and or safety of ICIs; nivolumab, pembrolizumab and ipilimumab. Review articles, case reports, editorials, and studies that used other combinations were excluded for this MA.

### Literature search and study selection

Two researchers (TTW, JP) conducted an independent literature search using healthcare electronic databases (PubMed, Embase and EbscoHost), Cochrane library and ClinicalKey. The articles obtained from the literature search were assessed by two researchers (TTW, JP) independently and disagreements were initially discussed. If an agreement was not reached, two researchers discussed with a third researcher (SNA) to mediate and finalize. The articles were screened according to the PRISMA flowchart. For the first stage, related articles were identified and grouped to create a total number of records. These records were screened to remove the review articles and non-relevant articles. The articles that did not meet the inclusion criteria based on abstract and title alone were excluded. Finally, full-text articles were examined to obtain the included studies required for MA.

### Data extraction

Two researchers (TTW, JP) independently extracted the relevant data from the included studies using piloted data extraction sheet. Discrepancies were discussed thoroughly and finalized with a third researcher (SNA). The extracted data included study title, first author with the year of publication, country, comparison, trial phase, site of malignant melanoma, number of patients, the ratio of male to female patients, number of patients given nivolumab alone, number of patients given ipilimumab alone and number of patients given combination therapy. Outcome data included were OS, PFS, ORR, any AEs, high-grade, haematological, gastrointestinal, dermatological, pulmonary, liver and endocrine AEs.

### Quality Assessment of the included studies

The Cochrane collaboration risk of bias assessment tool was used to assess the risk of bias in the included studies. This included the following assessment scopes: random sequence generation, allocation concealment, the blindness of participants and personnel, the blindness of outcome assessment, incomplete outcome data, selective reporting, and other biases. We rated each domain of the tool as 'low', 'high', or 'unclear' risk of bias at the study level and for each outcome 20.

### Statistical analysis

Among the outcomes, analysis of OS and PFS was estimated as the hazard ratio (HR) and analysis of other outcomes was estimated as the risk ratios (RR) for the treatment success of monotherapy versus combination ICIs. From the statistical analysis, heterogeneities were assessed using the chi-squared test and the I² statistic. Pearson's chi-squared test was used to decide a statistically significant difference between the expected frequencies and the observed frequencies. To avoid heterogeneity, if the I² statistic was more than 50%, a random-effects model was used; and if the I² statistic was less than 50%, a fixed-effect model was used. We investigated the robustness of the review by performing sensitivity analyses when appropriate, such as performing fixed-effect models for selected outcomes and including only 'low risk of bias' outcomes, according to the summary assessment of the risk of bias. A two-tailed P value of less than 0.05 was considered statistically significant. A 95% CI was used to provide a range of values for the ORs obtained. MA was performed with Review Manager (RevMan 5.4) software.

## Results

### Literature search results

**Figure 1:**
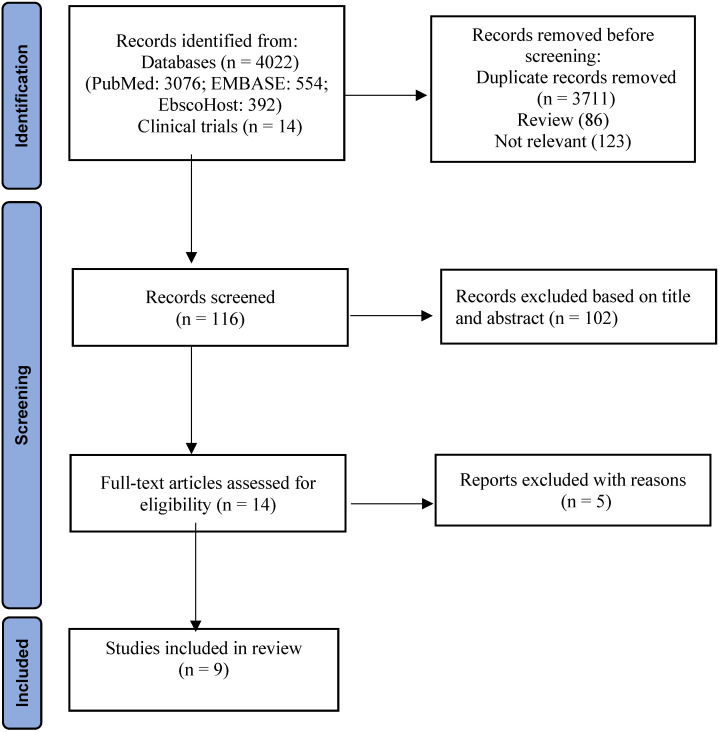
PRISMA flowchart indicating study selection 19.

A total of 4036 potential studies were identified using the preliminary search strategy; 3076 were from PubMed, 554 were from EMBASE, 392 were from EbscoHost and 14 were clinical trials from ClinicalKey. A total of 325 studies were compiled after removing the duplicates. Based on the titles, 86 review articles and 123 irrelevant studies were removed. After screening abstracts of the remaining 116 studies, 102 studies were excluded as they were not specifically related to specific search criteria. Finally, 14 studies were accessed for eligibility by reading the full text. Among them, five studies were excluded with reasons based on inclusion and exclusion criteria. Therefore, the remaining nine articles were included for SR and MA. A three-phase flow chart of the study selection process based on the updated PRISMA statement 2020 is illustrated in Figure 1. A summary of the reasons for five excluded studies 21-25 is shown in Table S1. PubMed search string was (((((((immune checkpoint inhibitors) OR immunotherapy) OR ipilimumab) OR nivolumab) OR pembrolizumab) AND monotherapy) AND combination) AND melanoma.

### Characteristics of the included studies

The characteristics of each included study were shown in Table 1. Nine RCT studies published from 2015 to 2021 were included in this MA. Among them, eight were published in peer-reviewed journals 26-33 and one was a clinical trial that updated the results in February 2021 34. All these nine studies included a total of 4150 participants, comparing combined nivolumab and ipilimumab; and nivolumab or ipilimumab alone. However, combination with pembrolizumab was not reported in all the included studies. Five studies were phase 2 RCTs and 4 were phase 3 RCTs.

### Assessment of risk of bias and publication bias

The results of the assessment of risk of bias are shown in the risk of bias graph (Figure 2A) and the risk of bias summary (Figure 2B). The overall risk of bias was evaluated as low risk and the quality of the studies was acceptable. Publication bias was assessed in nine included studies. Begg's and Egger's test Funnel plot showed some degree of publication bias, especially for the studies with a small sample size (Figure S1).

### Efficacy of combination Vs monotherapy of ICIs

**Figure 3:**
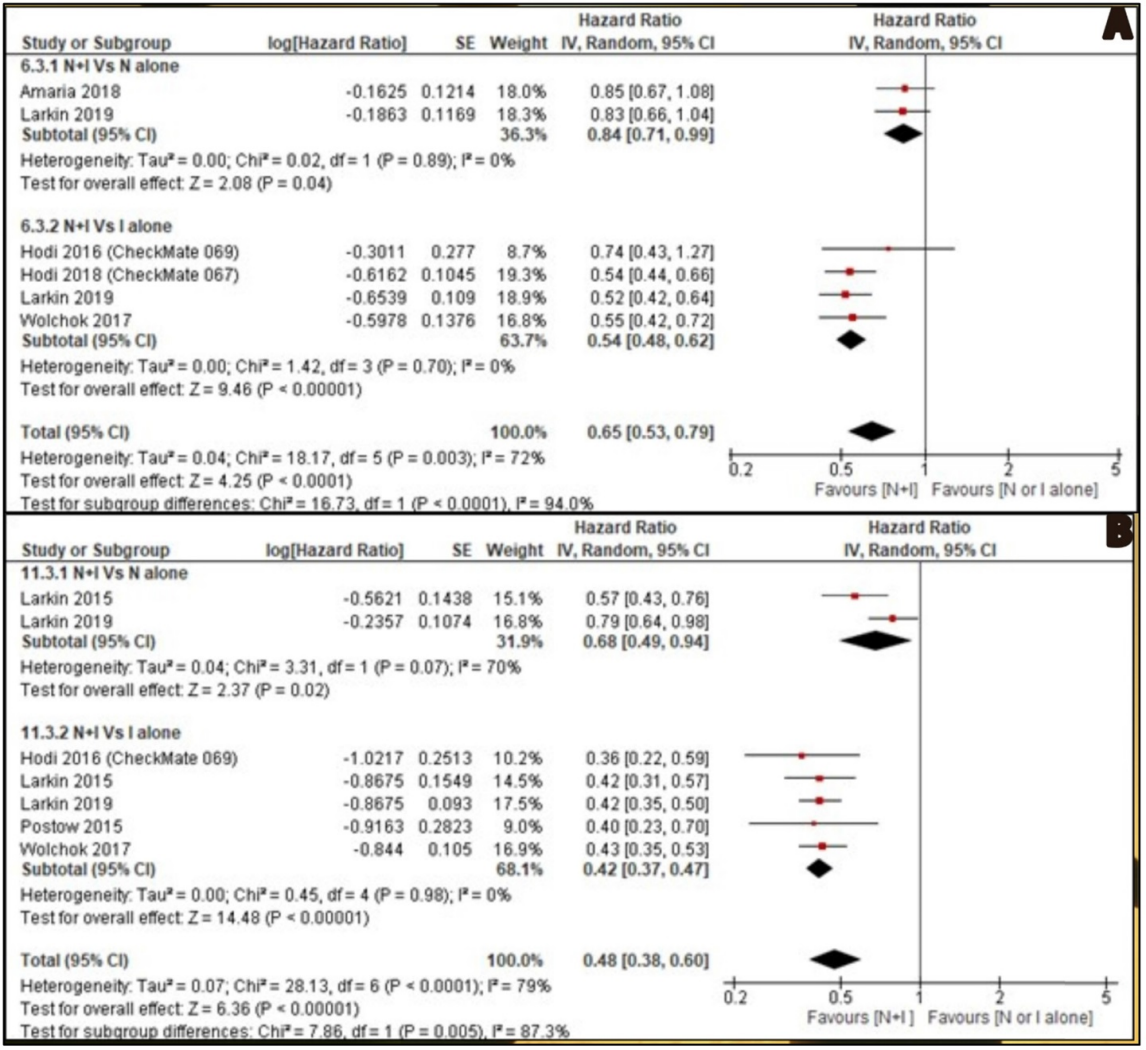
** A:** Forest plot for overall survival (OS); **B:** Forest plot for progression free survival (PFS).

OS was reported by six included studies. Two studies 26, 30 reported combined nivolumab and ipilimumab Vs nivolumab alone and four studies 27, 28, 30, 32 reported combined nivolumab and ipilimumab Vs ipilimumab alone. OS of combination therapy was better than monotherapy with HR 0.65, 95% CI [0.53, 0.79] and it was statistically significant (p<0.0001). Subgroup analysis also showed combination therapy had significant favourable OS compared with monotherapy, nivolumab alone or ipilimumab alone with HR 0.84, 95% CI [0.71, 0.99], p 0.04 and HR 0.54, 95% CI [0.48, 0.62], p <0.00001 respectively (Figure 3A).

PFS was reported by seven studies. Two studies 29, 30 reported combined nivolumab and ipilimumab Vs nivolumab alone and five studies 27, 29-32 reported combined nivolumab and ipilimumab Vs ipilimumab alone. PFS of combination therapy was better than monotherapy with HR 0.48, 95% CI [0.38, 0.60] and it was statistically significant (p<0.0001). Subgroup analysis also showed combination therapy had significant favourable PFS compared with monotherapy, nivolumab alone or ipilimumab alone with HR 0.68, 95% CI [0.49, 0.94], p 0.02 and HR 0.42, 95% CI [0.37, 0.47], p <0.00001 respectively (Figure 3B).

**Figure 4:**
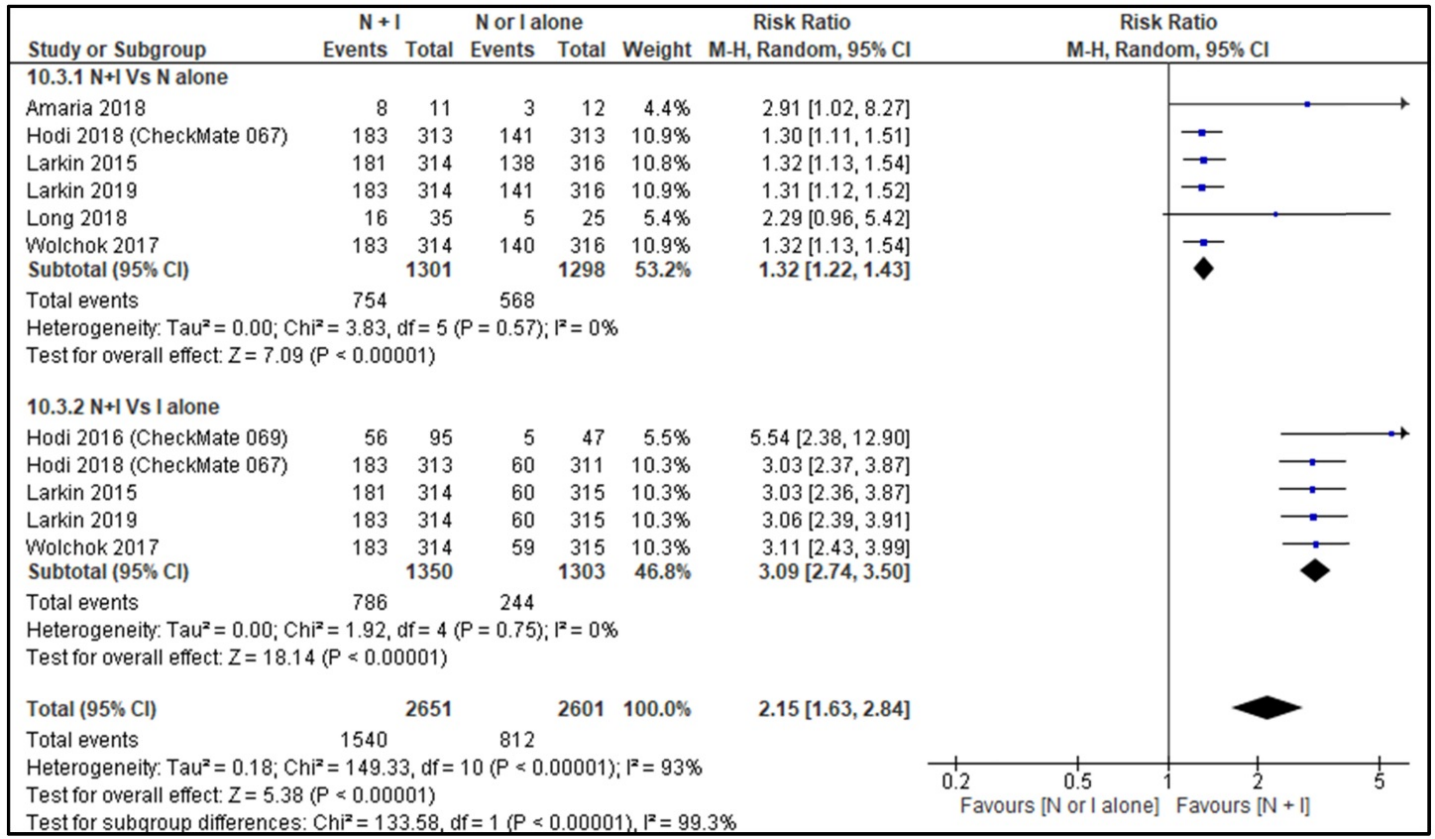
Forest plot for objective response rate (ORR).

ORR between combination therapy Vs nivolumab alone was reported by six studies 26, 28-30, 32, 33 and ORR between combination therapy Vs ipilimumab alone was reported by five studies 27-30, 32. ORR of combination therapy was better than monotherapy with RR 2.15, 95% CI [1.63, 2.84] and it was statistically significant (p=<0.00001). Subgroup analysis also showed combination therapy had significant favourable ORR compared with monotherapy, nivolumab alone or ipilimumab alone with HR 1.32, 95% CI [1.22, 1.43], p <0.00001 and HR 3.09, 95% CI [2.74, 3.50], p <0.00001 respectively (Figure 4).

As the given data regarding PD-1/PD-L1 expression status, prior chemotherapy and dose of ICIs were not sufficiently given by primary included studies, we could not manage to perform subgroup analysis for those confounding factors.

### Adverse effects of combination vs monotherapy of ICIs

In this MA, any treatment-related AEs were described by eight included studies 26-33. High-grade AEs, gastrointestinal AEs, dermatological AEs, liver AEs and endocrine AEs were described by all nine included studies. Haematological AEs were reported by six included studies 26, 28, 30-32, 34 and pulmonary AEs were reported by seven included studies 26-28, 30-33.

Table 2 indicates the summary of the incidence of various AEs comparing monotherapy and combination therapy with RR (95% CI) and p-value. There was reduced all types of AEs with monotherapy compared with combination therapy and all AEs comparison were statistically significant with a p-value less than 0.05.

**Figure 5:**
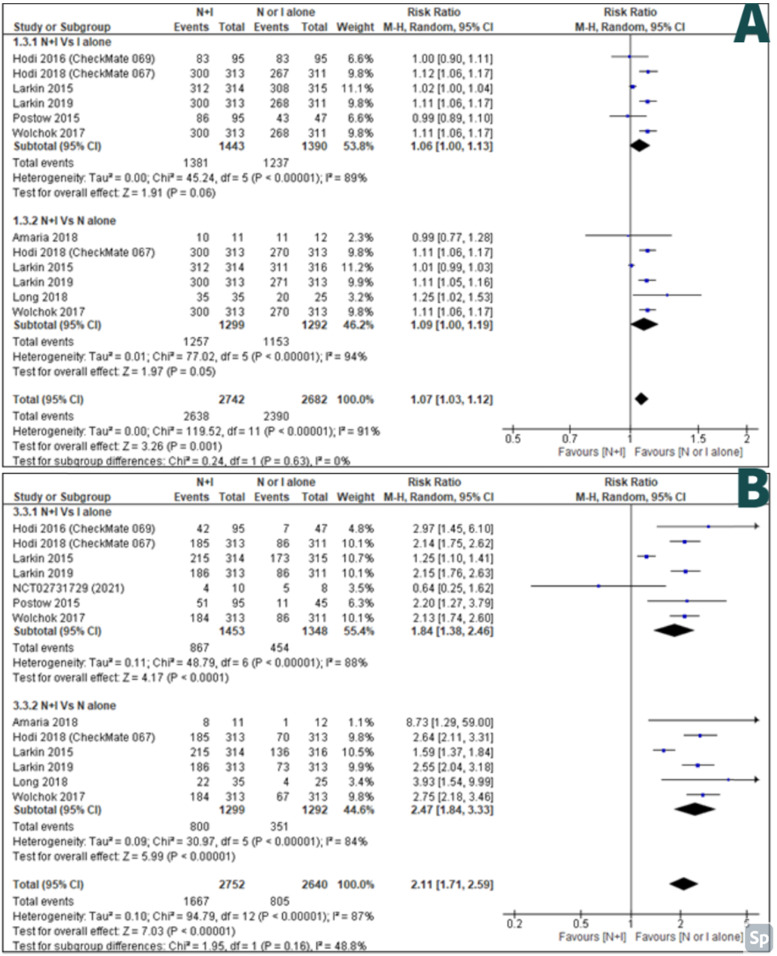
**A:** Forest plot for adverse effects of any grade; **B:** Forest plot for high grade adverse effects.

AEs of any grade between combination therapy Vs ipilimumab alone were reported by six studies 27-32 and AEs of any grade between combination therapy Vs nivolumab alone was reported by six studies 26, 28-30, 32, 33. There were less AEs of any grade in monotherapy than combination therapy; with RR 1.07, 95% CI [1.03-1.12] and it was statistically significant (p=<0.001) (Figure 5A).

High-grade AEs between combination therapy Vs ipilimumab alone were reported by seven studies 27-32, 34 and high-grade AEs between combination therapy Vs nivolumab alone were reported by six studies 26, 28-30, 32, 33. There were less high-grade AEs in monotherapy than combination therapy; with RR 2.11, 95% CI [1.71-2.59] and it was statistically significant (p=<0.00001) (Figure 5B).

Haematological AEs between combination therapy Vs ipilimumab alone were reported by four studies 28, 30-32 and combination therapy Vs nivolumab alone were reported by five studies 26, 28, 29, 32, 34. There were reduced haematological AEs in monotherapy than combination therapy; with RR 1.43, 95% CI [1.03-1.97] and it was statistically significant (p=<0.03). However, combination therapy Vs ipilimumab alone did not show a statistically significant difference for Haematological AEs (p=0.89) (Figure S2).

Gastrointestinal AEs between combination therapy Vs ipilimumab alone were reported by six studies 27-32 and combination therapy Vs nivolumab alone were reported by seven studies 26, 28-30, 32-34. There were reduced gastrointestinal AEs in monotherapy than combination therapy; with RR 1.69, 95% CI [1.39, 2.06] and it was statistically significant (p=<0.00001) (Figure S3).

Dermatological AEs between combination therapy Vs ipilimumab alone were reported by six studies 27-32 and combination therapy Vs nivolumab alone were reported by seven studies 26, 28-30, 32-34. There were reduced dermatological AEs in monotherapy than combination therapy; with RR 1.32, 95% CI [1.19, 1.46] and it was statistically significant (p=<0.00001) (Figure S4).

Pulmonary AEs between combination therapy Vs ipilimumab alone were reported by five studies 28, 29, 30-32 and combination therapy Vs nivolumab alone were reported by five studies 26, 28, 30, 32, 33. There were reduced pulmonary AEs in monotherapy than combination therapy; with RR 4.25, 95% CI [2.97, 6.10] and it was statistically significant (p=<0.00001) (Figure S5).

Liver AEs between combination therapy Vs ipilimumab alone were reported by six studies 27-32 and combination therapy Vs nivolumab alone were reported by six studies 26, 28-30, 32, 33. There were reduced liver AEs in monotherapy than combination therapy; with RR 4.36, 95% CI [3.76, 5.06] and it was statistically significant (p=<0.00001) (Figure S6).

Endocrine AEs between combination therapy Vs ipilimumab alone were reported by six studies 27-32 and combination therapy Vs nivolumab alone were reported by seven studies 26, 28-30, 32-34. There were reduced endocrine AEs in monotherapy than combination therapy; with RR 2.63, 95% CI [2.16, 3.21] and it was statistically significant (p=<0.00001) (Figure S7).

## Discussion

In recent years, the role of immunotherapy in cancer treatment has expanded and now it is the first choice of treatment intervention in many cancers 35. Both in-vivo and clinical studies of combined chemotherapy and ICIs showed promising results in many solid tumours such as renal cell carcinoma and non-small cell lung carcinoma 36, 37. Immunotherapeutic drugs are used as single ICI or combined ICIs or combined with chemotherapeutic drugs. A Cochrane systematic review on systematic treatment for metastatic cutaneous MM reported that PD-1 inhibitors (nivolumab and pembrolizumab) improved OS and PFS compared to chemotherapy and CTLA-4 inhibitors (ipilimumab and tremelimumab) 38.

In the treatment of many solid cancers such as non-small cell lung cancers, oesophageal carcinoma and malignant mesothelioma, the efficacy of combined ICIs is better than single ICI 39. Combined two ICIs are the most promising approaches in the treatment of advanced MM 40. The majority of combination ICIs used are CTLA-4 inhibitors (ipilimumab) and PD-1 inhibitors (nivolumab or pembrolizumab). These drugs have been authorized by the United States Food and Drug Administration (FDA) for the treatment of advanced MM in 2011 and 2014 41-43.

In this MA, we only compared the combination therapy of nivolumab and ipilimumab with either nivolumab or ipilimumab alone as included primary studies reported this combination only. OS showed that there was a statistically significant difference between combined nivolumab and ipilimumab with either nivolumab or ipilimumab alone. Therefore, OS of combination ICIs therapy was better than monotherapy. It also showed a clear statistically significant difference in the PFS between combined nivolumab and ipilimumab with either nivolumab or ipilimumab alone. Therefore, PFS of combination ICIs therapy was better than monotherapy. ORR of combination ICIs therapy was also better than that of monotherapy significantly. These results on OS and PFS are concordant with the results of a Cochrane systematic review which studied the outcomes of combined ICIs in the treatment of advanced non-small cell lung cancer. However, combined ICIs did not improve PFS and ORR compared to platinum-based chemotherapy in that study 44.

Our MA showed that combination therapy improved OS, PFS and ORR compared to nivolumab alone or ipilimumab alone, and this finding is concordant with the results of the other two MAs on the efficacy of ICIs in various types of cancers 17, 39. Another MA on the efficacy of combined ICIs also reported that improved ORR was seen in the combined nivolumab and ipilimumab treatment group in advanced MM 18. A phase 2 RCT that studied the effects of combined nivolumab and ipilimumab in advanced MM metastasized to the brain also reported clinically meaningful efficacy with combined ICIs therapy 45. Among the targeted therapies of metastatic MM, combination ICIs had clinically significant intracranial efficacy 46. It was also reported that improved ORR and better response rate were seen with combined nivolumab and ipilimumab therapy in various types of solid cancers including MM 39. This might be due to nivolumab which is PD-1 inhibitor, and it was reported that anti-PD1 monoclonal antibodies are better than anti‐CTLA4 monoclonal antibodies in terms of OS in the treatment of metastatic cutaneous MM 38. Another MA on ICIs in metastatic mucosal MM also reported that monoclonal antibodies targeting the PD-1 and PD-L1 interaction seemed to be more effective than targeting CTLA-4 in the treatment of MM 47. In the treatment of many solid tumours, combined antibodies of PD-1 and PD-L1 have been widely used and it provides better results than anti-CTLA4 therapy 48.

In this MA, we could not manage to analyze complete and partial responses as some of the included studies reported only ORR without specific data on complete and partial response rates. Some included studies reported OS and PFS based on the expression of PD-1 and PD-L1 by the tumour cells. As not all included studies did not report those expressions, analyses based on those expressions were not included in this MA. Among nine included studies, two studies reported that both combination ICIs therapy and nivolumab monotherapy showed improved OS, ORR and PFS regardless of PD-L1 expression 29, 32. A MA of ICIs on non-small cell carcinoma reported that combined ICIs prolonged OS compared to platinum-based chemotherapy in people with PD-L1 expression ≥50% 44.

Many combination ICIs have been developed in the treatment of solid tumours, especially MM, and nivolumab plus ipilimumab is the most used combination 49. However, nivolumab is a PD-1 inhibitor and ipilimumab is a CTLA-4 inhibitor, their mechanisms of action are not the same and not complementary to each other 50. It is assumed that antitumor activity of CTLA-4 inhibitors may be enhanced by tumour-specific T effector cells suppression through the PD-L1/PD-1 pathway and that immune suppression is partially mediated by CTLA-4 inhibitors itself 51. The efficacy of PD-1 inhibitors might be compromised by the lack of full activation of tumour-specific effector T cells mediated by CTLA-4, which is also thought to be aggravated by upregulation of CTLA-4 induced by the PD-1 inhibition itself 50, 52. Therefore, the higher immune-mediated anti-tumour activity gave better efficacy with nivolumab plus ipilimumab than nivolumab or ipilimumab alone in the treatment of advanced MM and non-small cell lung carcinoma 53. The success of cancer treatment with ICIs is not only due to targeting cancer cell destruction through the activation of the host immune system, but it also targets the cancer-immune environment with activation of tumour infiltrating T lymphocytes 54.

Another ICI used in the treatment of MM is relatilimab which block the lymphocyte-activation gene 3 (LAG-3) expressed on immune cells including T cells, and negatively regulates T-cell proliferation and effector T-cell function. A clinical trial (NCT03470922) has been done on efficacy of combine relatilimab and nivolumab 55, and it showed better PFS although it showed no new safety signals 56. As there is only one clinical trial on it, we did not include the efficacy and safety regarding combination with relatilimab in this MA.

ICIs can cause immune-related AEs as the mechanism of ICI action relies on the inhibition of the physiological brake of immune activation and they often have off-target effects resulting in immune-mediated inflammation of diverse organs or tissues 54. The spectrum of AEs caused by ICIs is different from those of chemotherapy and they are mainly autoimmune and autoinflammatory complications 57. CTLA-4 inhibitors carry a higher risk and severity of immune-related AEs compared to other ICIs 58. The potential pathophysiological mechanisms involved in the development of immune-related AEs are T cell-mediated mechanism, B cell-mediated effects, CTLA-4 expression in the tissue and inflammatory cytokine-mediated mechanism 59.

In this MA, we analyzed common treatment-related AEs caused by a single ICI either nivolumab or ipilimumab alone and combined nivolumab plus ipilimumab. The incidence of AEs analyzed in this MA were any treatment-related AEs, high-grade AEs, haematological, gastrointestinal, dermatological, pulmonary, liver and endocrine AEs. It showed that a single ICI either nivolumab or ipilimumab alone reduced all types of AEs compared to combined nivolumab plus ipilimumab, and it showed statistically significant results for all AEs. Therefore, monotherapy either nivolumab or ipilimumab alone is safer than combination ICIs therapy. This finding is concordant with the result reported by a MA that studied potential immune-related AEs in monotherapy and combination therapy of ipilimumab, nivolumab, and pembrolizumab in advanced MM 60. Another MA that studied the safety of combination ICIs therapy in advanced solid tumours also reported that severe AEs were associated with combination ICIs compared with monotherapy 17. A MA of ICIs in non-small cell lung cancer which compared ICIs and platinum-based chemotherapy reported that high-grade AEs were rare with single ICI compared to chemotherapy; however, the frequency of those AEs was not significantly different between combination ICIs and chemotherapy 44. In the treatment of metastatic cutaneous MM, although anti-PD1 monoclonal antibodies are the least toxic regimen, combination ICIs increase toxicity compared to chemotherapy and other targeted therapies such as BRAF inhibitors 38.

Most of the studies in our MA reported grade 3 and 4 AEs as high-grade AEs. No grade 5 AEs resulting in death were reported in all included studies. The most common haematological AEs were anaemia, leucopenia and thrombocytopenia. The most common gastrointestinal AEs were vomiting, diarrhoea and colitis. The most common dermatological AEs reported were skin rash and pruritis. Pulmonary AEs were reported as pneumonia or pneumonitis. Liver AEs included increased serum levels of alanine transaminases and aspartate transaminases. Endocrine AEs included hyperthyroidism, hypothyroidism and hypophysitis/ hypopituitarism, and only a few reported hyperglycaemia/ diabetes mellitus. Most of the treatment-related AEs developed within a month of the last dose in nivolumab plus ipilimumab group and the majority were resolved as they were not severe and manageable 61. A MA that studied different doses of nivolumab and ipilimumab in the combination group found that AEs were slightly increased in the group with nivolumab 1 mg/kg plus ipilimumab 3 mg/kg compared with nivolumab 3 mg/kg plus ipilimumab 1 mg/kg 39. In our MA, many include studies did not specifically mention the dose of ICIs.

CTLA-4 inhibitor (ipilimumab) was mostly associated with an increased incidence of gastrointestinal, renal, dermatological, and endocrine AEs. The AEs of PD-1 inhibitors (nivolumab or pembrolizumab) were the endocrine, gastrointestinal, liver, musculoskeletal, nervous system, renal, and respiratory AEs 60. Cessation of the ICIs, initiation of steroids and supportive therapy were the preferred choice of treatment for those AEs 62, 63. A MA showed toxicity-related fatality rates of 0.36% (anti-PD-1), 0.38% (anti-PD-L1), 1.08% (anti-CTLA-4), and 1.23% (PD-1/PD-L1 plus CTLA-4). Although rare, ICI-related deaths may occur when severe iatrogenic AEs such as myocarditis, encephalitis, or acute hypophysitis are not readily diagnosed and these AEs were treated with high dose steroids and more potent immunosuppressors 64. Most of the included studies in our MA reported that most treatment-related AEs were generally manageable, and no study reported death from AEs. Various biomarkers are known to be associated with the onset of immune-related AEs caused by ICIs. Although most of these biomarkers such as T cell, B cells, microbiome and genomic biomarkers are not bona fide predictive markers, some of them have potential clinical utility 65. The autoimmune status of the patient should be checked before choosing ICI for the treatment.

This MA had few limitations in conducting data extraction and MA of those data. For efficacy and safety, different included studies used different follow-up duration. If there was a standardized follow-up duration, data and results especially on efficacy will be more accurate. Another limitation was that our MA was based on unadjusted data analysis, and we could not manage to analyze based on other confounding factors such as age, sex, PD-1/PD-L1 expression, prior chemotherapy and dose of ICIs etc. Another limitation was that the studies on combination with relatlimab, anti- LAG-3 were not included in this MA as enough number of primary studies to perform MA have not been published.

In conclusion, this MA showed that the efficacy of the combined ICIs was more favourable than single ICI in terms of OS, PFS and ORR. There was a significant difference in the combination Vs monotherapy in terms of OS, PFS and ORR. Although combination ICIs therapy showed better efficacy than monotherapy, monotherapy (either nivolumab or ipilimumab) was safer than combination therapy as it tended to decrease the incidence of most of the treatment-related AEs. Taking the patient's safety as our main priority, the most appropriate clinical practice would be prescribing monotherapy over combination therapy in the treatment of advanced MM. Starting with monotherapy using a single ICI followed by adding another ICI can be considered to minimize the AEs and improve efficacy in the treatment of advanced MM. Studies on how immune-related AEs can be separated from the anti-tumour effects of ICIs and the identification of specific biomarkers to predict the development of the toxicities need to be thoroughly undertaken for the improvement of patient outcomes.

## Supplementary Material

Supplementary figures.

## Figures and Tables

**Figure 2A:**
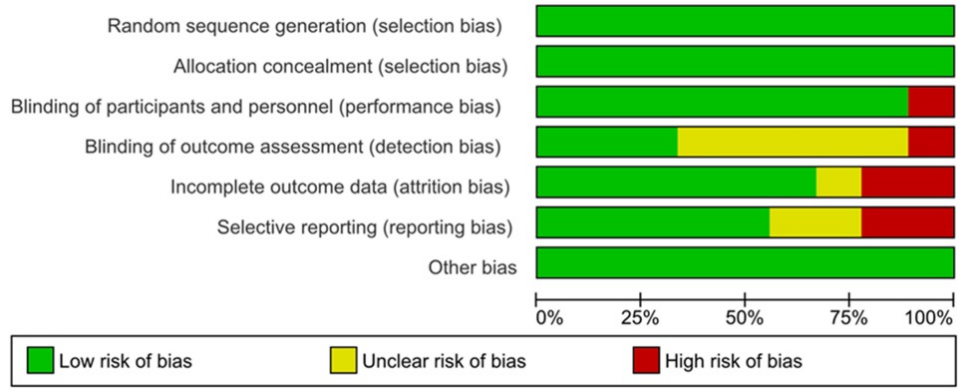
Assessment of risk of bias (Risk of Bias Graph).

**Figure 2B:**
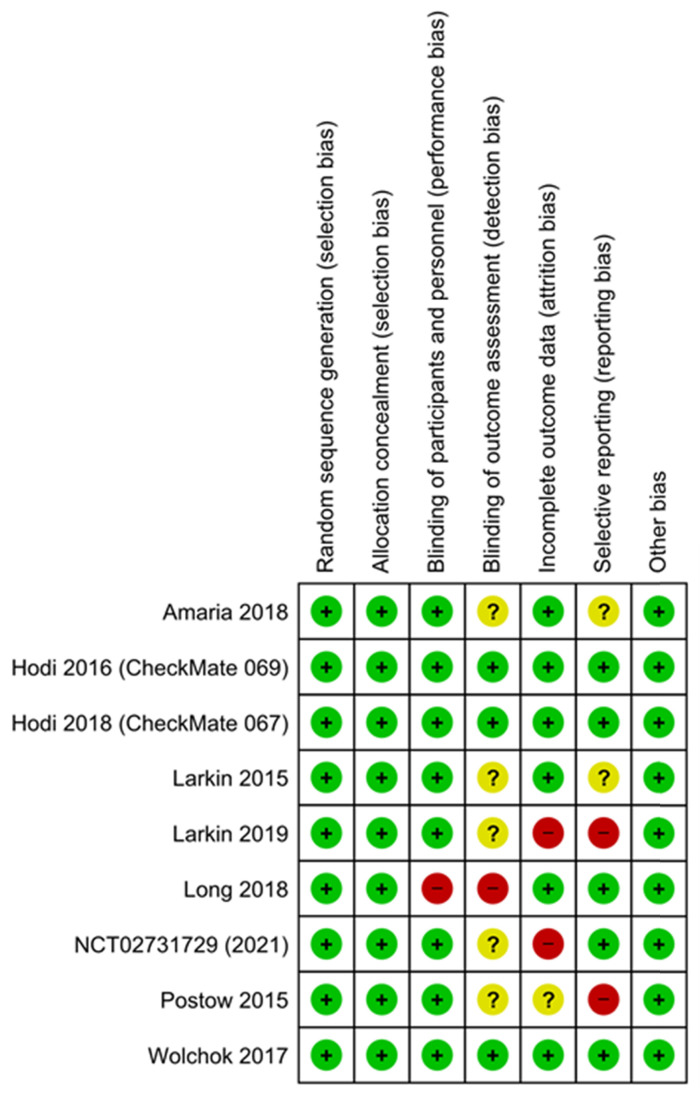
Assessment of risk of bias (Risk of Bias summary).

**Table 1:** Characteristics of the included studies

Study, Year Trial identifier (Reference)	Country	Comparison	Trial phase	Site of MM	No: of patient	M/F	N alone	I alone	Combined N + I
Amaria 2018 NCT02519322 26	US	N+I Vs N alone	2	All	23	19/4	12	-	11
Hodi 2016 (CheckMate 069),NCT01927419 27	France, US	N+I Vs I alone	2	skin, mucosa	142	95/47	-	47	95
Hodi 2018 (CheckMate 067),NCT01844505 28	France, US	N+I Vs I alone	3	skin, mucosa	937	605/332	313	311	313
		N+I Vs N alone							
Larkin 2015NCT01844505 29	US, Europe, Asia, Africa	N+I Vs I alone	3	All	945	610/335	316	315	314
		N+I Vs N alone							
Larkin 2019 NCT01844505 30	US, Europe, Asia, Africa	N+I Vs I alone	3	All	937	605/332	313	311	313
		N+I Vs N alone							
Postow 2015 NCT01927419 31	US, Europe	N+I Vs I alone	2	All	142	95/47	-	47	95
Wolchok 2017 NCT01844505 32	US, Europe	N+I Vs I alone	3	All	945	610/335	316	315	314
		N+I Vs N alone							
Long 2018 NCT02374242 33	Australia	N+I Vs N alone	2	MM with brain metastasis	60	48/12	25	-	35
NCT02731729 (2021) 34	US	N+I Vs I alone	2	skin, mucosa	19	15/4	-	9	10

N: Nivolumab; I: Ipilimumab

**Table 2:** Incidence of various adverse effects comparing monotherapy and combination therapy.

Adverse Effects (AEs)	No. of studies	RR (95% CI)	*p-*value	*I^2^*	Statistical method
Any AEs	8	1.07 [1.03-1.12]	< 0.001	91%	Random
High grade AEs	9	2.11 [1.71-2.59]	< 0.00001	87%	Random
Haematological AEs	6	1.43 [1.03-1.97]	0.03	61%	Random
Gastrointestinal AEs	9	1.69 [1.39-2.06]	< 0.00001	88%	Random
Dermatological AEs	9	1.32 [1.19-1.46]	< 0.00001	78%	Random
Pulmonary AEs	7	4.25[2.97-6.10]	< 0.00001	0%	Fixed
Liver AEs	9	4.36 [3.76 -5.06]	< 0.00001	0%	Fixed
Endocrine AEs	9	2.63 [2.16-3.21]	< 0.00001	52%	Random
