# Supplementary material for: Efficacy and Safety of Immune Checkpoint Inhibitors for Advanced Malignant Melanoma: A Meta-Analysis on Monotherapy Vs Combination Therapy
Source: J Cancer. 2022 Aug 8;13(10):3091–102. doi: 10.7150/jca.72210 (PMC9414012; doi:10.7150/jca.72210)
Supplement: Supplementary file 1 — Supplementary figures. [file jcav13p3091s1.pdf]

Study or Subgroup	N+I Events	N+I Total	N or I alone Events	N or I alone Total	Weight	Risk Ratio M-H, Random, 95% CI
**2.3.1 N+I Vs I alone**						
Hodi 2018 (CheckMate 067)	38	313	38	311	15.2%	0.99 [0.65, 1.51]
Larkin 2019	38	313	38	311	15.2%	0.99 [0.65, 1.51]
Postow 2015	15	95	8	45	9.3%	0.89 [0.41, 1.94]
Wolchok 2017	38	313	38	311	15.2%	0.99 [0.65, 1.51]
**Subtotal (95% CI)**		**1034**		**978**	**54.9%**	**0.98 [0.78, 1.24]**
Total events	129		122			
Heterogeneity: $Tau^2 = 0.00$; $Chi^2 = 0.07$, df = 3 (P = 0.99); $I^2 = 0\%$						
Test for overall effect: Z = 0.14 (P = 0.89)						
**2.3.2 N+I Vs N alone**						
Amaria 2018	3	11	1	12	2.1%	3.27 [0.40, 27.00]
Hodi 2018 (CheckMate 067)	38	313	16	313	12.6%	2.38 [1.35, 4.17]
Larkin 2019	38	313	16	313	12.6%	2.38 [1.35, 4.17]
NCT02731729 (2021)	4	10	3	9	5.4%	1.20 [0.36, 3.97]
Wolchok 2017	38	313	15	313	12.4%	2.53 [1.42, 4.51]
**Subtotal (95% CI)**		**960**		**960**	**45.1%**	**2.33 [1.70, 3.18]**
Total events	121		51			
Heterogeneity: $Tau^2 = 0.00$; $Chi^2 = 1.38$, df = 4 (P = 0.85); $I^2 = 0\%$						
Test for overall effect: Z = 5.30 (P < 0.00001)						
**Total (95% CI)**		**1994**		**1938**	**100.0%**	**1.43 [1.03, 1.97]**
Total events	250		173			
Heterogeneity: $Tau^2 = 0.13$; $Chi^2 = 20.36$, df = 8 (P = 0.009); $I^2 = 61\%$						
Test for overall effect: Z = 2.15 (P = 0.03)						
Test for subgroup differences: $Chi^2 = 18.78$, df = 1 (P < 0.0001), $I^2 = 94.7\%$						

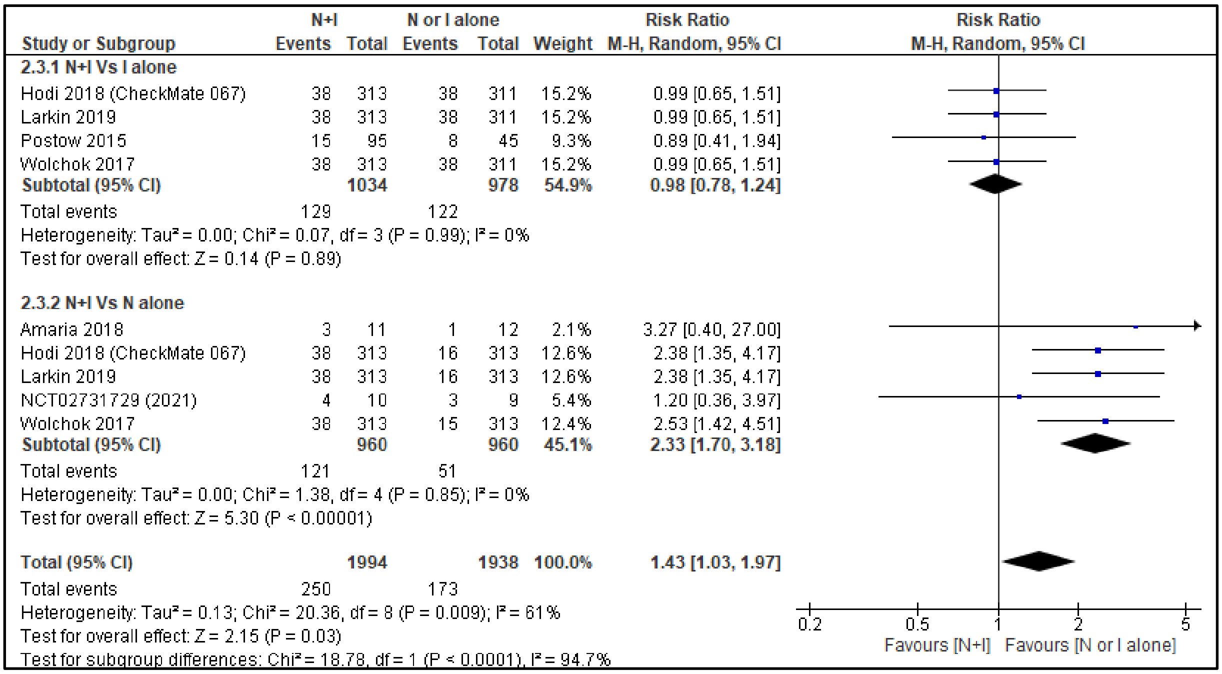

Figure S2: Forest plot for haematological adverse effects.

Study or Subgroup	N+I Events	N+I Total	N or I alone Events	N or I alone Total	Weight	Risk Ratio M-H, Random, 95% CI
**4.3.1 N+I Vs I alone**						
Hodi 2016 (CheckMate 069)	46	95	16	47	6.7%	1.42 [0.91, 2.23]
Hodi 2018 (CheckMate 067)	182	313	139	311	9.7%	1.30 [1.11, 1.52]
Larkin 2015	175	314	139	315	9.7%	1.26 [1.08, 1.48]
Larkin 2019	183	313	141	311	9.7%	1.29 [1.11, 1.50]
Postow 2015	64	95	23	45	8.1%	1.32 [0.96, 1.81]
Wolchok 2017	182	313	140	311	9.7%	1.29 [1.11, 1.51]
**Subtotal (95% CI)**		**1443**		**1340**	**53.6%**	**1.29 [1.20, 1.39]**
Total events	832		598			
Heterogeneity: $Tau^2 = 0.00$; $Chi^2 = 0.28$, df = 5 (P = 1.00); $I^2 = 0\%$						
Test for overall effect: Z = 6.74 (P < 0.00001)						
**4.3.2 N+I Vs N alone**						
Amaria 2018	9	11	1	12	1.0%	9.82 [1.47, 65.45]
Hodi 2018 (CheckMate 067)	182	313	77	313	9.2%	2.36 [1.91, 2.93]
Larkin 2015	175	314	64	316	8.9%	2.75 [2.16, 3.50]
Larkin 2019	183	313	78	313	9.2%	2.35 [1.89, 2.91]
Long 2018	35	35	15	25	8.1%	1.65 [1.20, 2.28]
NCT02731729 (2021)	1	10	3	9	0.8%	0.30 [0.04, 2.39]
Wolchok 2017	182	313	74	313	9.1%	2.46 [1.97, 3.07]
**Subtotal (95% CI)**		**1309**		**1301**	**46.4%**	**2.32 [1.95, 2.75]**
Total events	767		312			
Heterogeneity: $Tau^2 = 0.02$; $Chi^2 = 12.72$, df = 6 (P = 0.05); $I^2 = 53\%$						
Test for overall effect: Z = 9.57 (P < 0.00001)						
**Total (95% CI)**		**2752**		**2641**	**100.0%**	**1.69 [1.39, 2.06]**
Total events	1599		910			
Heterogeneity: $Tau^2 = 0.10$; $Chi^2 = 100.54$, df = 12 (P < 0.00001); $I^2 = 88\%$						
Test for overall effect: Z = 5.29 (P < 0.00001)						
Test for subgroup differences: $Chi^2 = 37.31$, df = 1 (P < 0.00001), $I^2 = 97.3\%$						

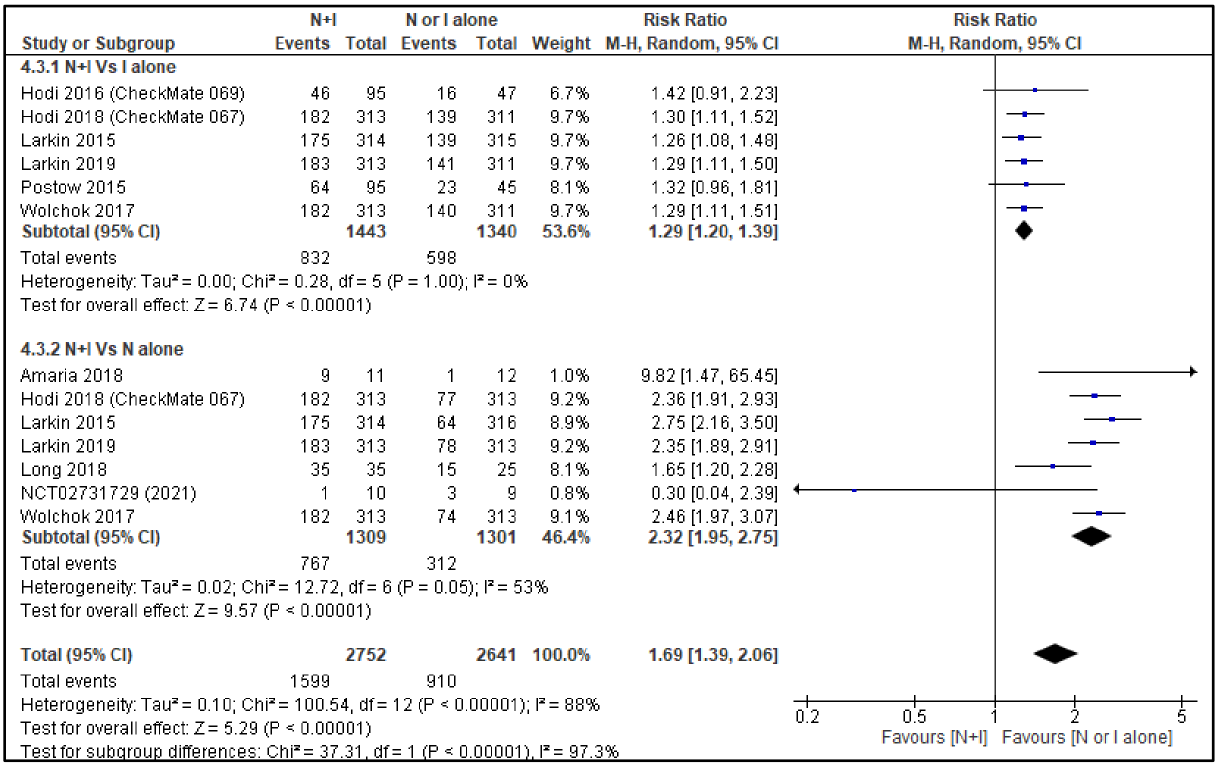

Figure S3: Forest plot for gastrointestinal adverse effects.

Study or Subgroup	N+I Events	N+I Total	N or I alone Events	N or I alone Total	Weight	Risk Ratio M-H, Random, 95% CI
**5.3.1 N+I Vs I alone**						
Hodi 2016 (CheckMate 069)	69	95	29	47	6.9%	1.18 [0.91, 1.52]
Hodi 2018 (CheckMate 067)	205	313	182	311	10.2%	1.12 [0.99, 1.27]
Larkin 2015	230	314	212	315	10.7%	1.09 [0.98, 1.21]
Larkin 2019	205	313	182	311	10.2%	1.12 [0.99, 1.27]
Postow 2015	72	95	25	45	6.3%	1.36 [1.03, 1.81]
Wolchok 2017	205	313	181	311	10.2%	1.13 [0.99, 1.27]
**Subtotal (95% CI)**		**1443**		**1340**	**54.7%**	**1.12 [1.06, 1.19]**
Total events	986		811			
Heterogeneity: Tau² = 0.00; Chi² = 2.30, df = 5 (P = 0.81); I² = 0%						
Test for overall effect: Z = 4.05 (P < 0.0001)						
**5.3.2 N+I Vs N alone**						
Amaria 2018	11	11	6	12	2.7%	1.92 [1.10, 3.34]
Hodi 2018 (CheckMate 067)	205	313	143	313	9.7%	1.43 [1.24, 1.66]
Larkin 2015	230	314	140	316	9.8%	1.65 [1.44, 1.90]
Larkin 2019	205	313	146	313	9.8%	1.40 [1.22, 1.62]
Long 2018	29	35	6	25	1.8%	3.45 [1.69, 7.05]
NCT02731729 (2021)	7	10	5	9	1.8%	1.26 [0.62, 2.57]
Wolchok 2017	205	313	139	313	9.6%	1.47 [1.27, 1.71]
**Subtotal (95% CI)**		**1309**		**1301**	**45.3%**	**1.52 [1.38, 1.68]**
Total events	892		585			
Heterogeneity: Tau² = 0.01; Chi² = 9.32, df = 6 (P = 0.16); I² = 36%						
Test for overall effect: Z = 8.25 (P < 0.00001)						
**Total (95% CI)**		**2752**		**2641**	**100.0%**	**1.32 [1.19, 1.46]**
Total events	1878		1396			
Heterogeneity: Tau² = 0.02; Chi² = 53.70, df = 12 (P < 0.00001); I² = 78%						
Test for overall effect: Z = 5.16 (P < 0.00001)						
Test for subgroup differences: Chi² = 27.12, df = 1 (P < 0.00001), I² = 96.3%						

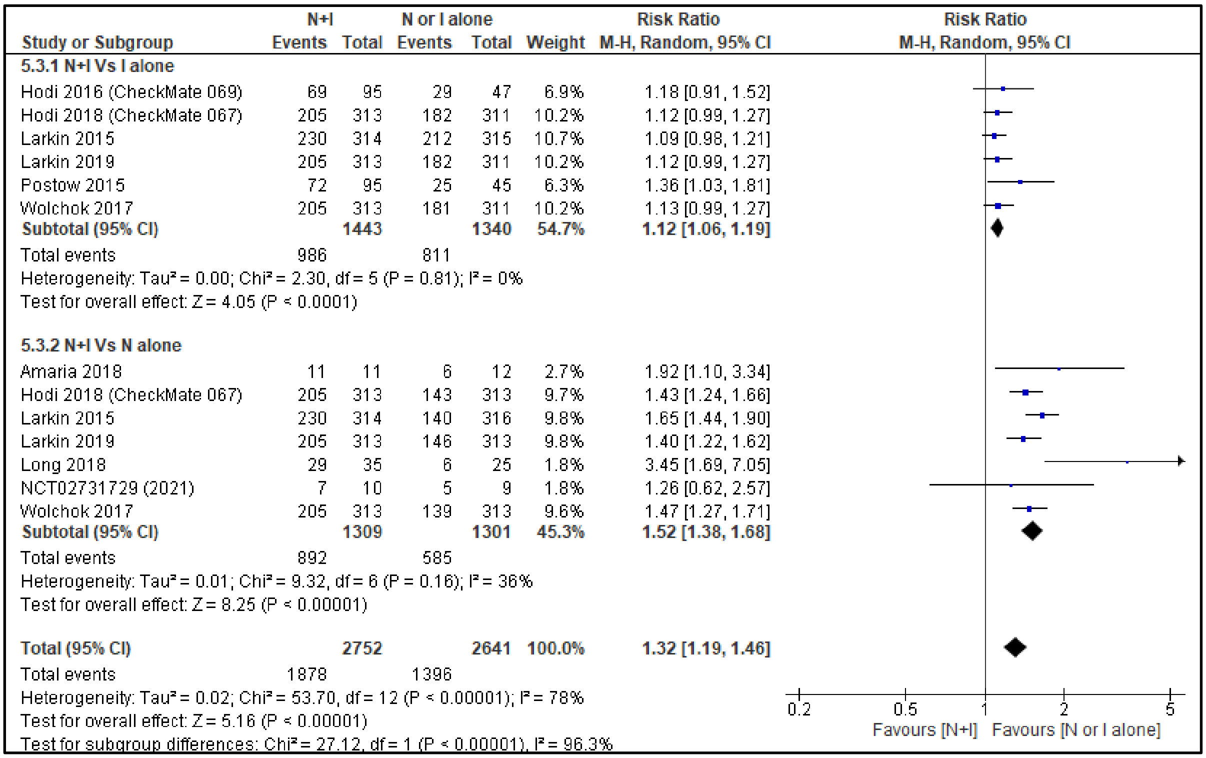

Figure S4: Forest plot for dermatological adverse effects.

Study or Subgroup	N+I Events	N+I Total	N or I alone Events	N or I alone Total	Weight	Risk Ratio M-H, Fixed, 95% CI
**7.3.1 N+I Vs I alone**						
Hodi 2016 (CheckMate 069)	10	95	1	47	3.7%	4.95 [0.65, 37.51]
Hodi 2018 (CheckMate 067)	23	313	5	311	13.8%	4.57 [1.76, 11.87]
Larkin 2019	23	313	5	311	13.8%	4.57 [1.76, 11.87]
Postow 2015	10	95	2	45	7.5%	2.37 [0.54, 10.36]
Wolchok 2017	22	313	5	311	13.8%	4.37 [1.68, 11.40]
**Subtotal (95% CI)**		**1129**		**1025**	**52.7%**	**4.23 [2.57, 6.97]**
Total events	88		18			
Heterogeneity: Chi² = 0.67, df = 4 (P = 0.95); I² = 0%						
Test for overall effect: Z = 5.66 (P < 0.00001)						
**7.3.2 N+I Vs N alone**						
Amaria 2018	1	11	1	12	2.6%	1.09 [0.08, 15.41]
Hodi 2018 (CheckMate 067)	23	313	5	313	13.8%	4.60 [1.77, 11.95]
Larkin 2019	23	313	5	313	13.8%	4.60 [1.77, 11.95]
Long 2018	5	35	1	25	3.2%	3.57 [0.44, 28.72]
Wolchok 2017	22	313	5	313	13.8%	4.40 [1.69, 11.47]
**Subtotal (95% CI)**		**985**		**976**	**47.3%**	**4.28 [2.54, 7.19]**
Total events	74		17			
Heterogeneity: Chi² = 1.10, df = 4 (P = 0.89); I² = 0%						
Test for overall effect: Z = 5.48 (P < 0.00001)						
**Total (95% CI)**		**2114**		**2001**	**100.0%**	**4.25 [2.97, 6.10]**
Total events	162		35			
Heterogeneity: Chi² = 1.77, df = 9 (P = 0.99); I² = 0%						
Test for overall effect: Z = 7.88 (P < 0.00001)						
Test for subgroup differences: Chi² = 0.00, df = 1 (P = 0.98), I² = 0%						

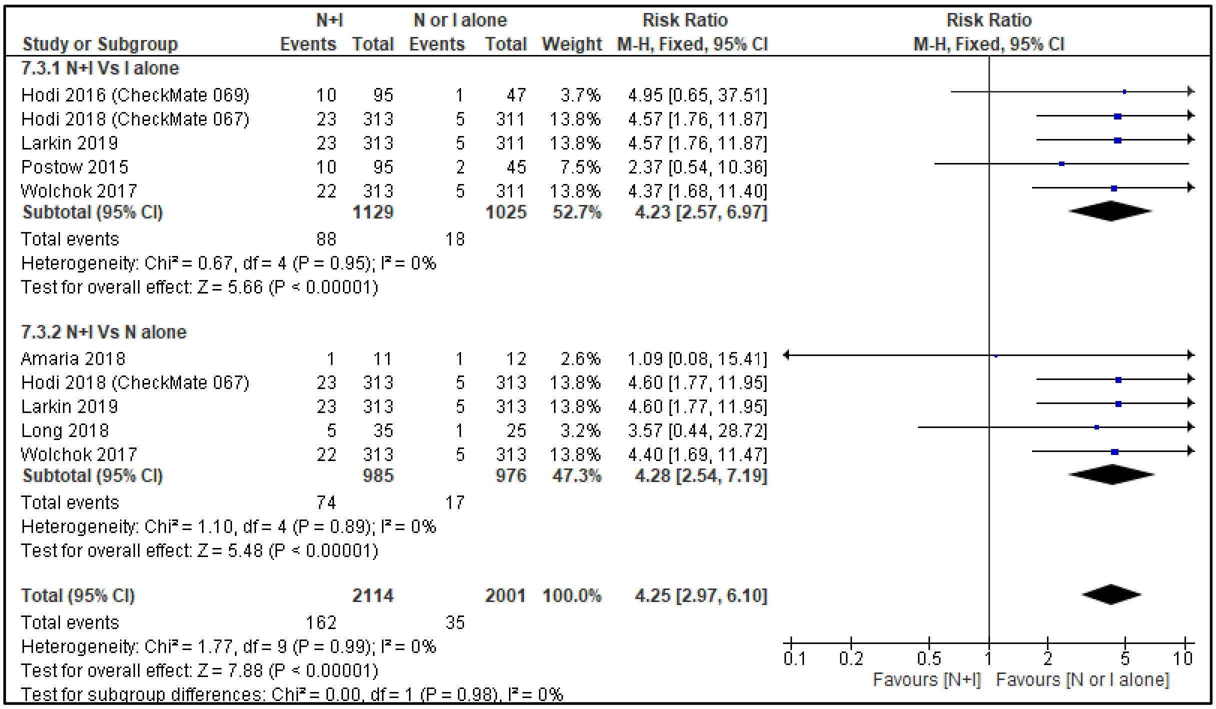

Figure S5: Forest plot for pulmonary adverse effects.

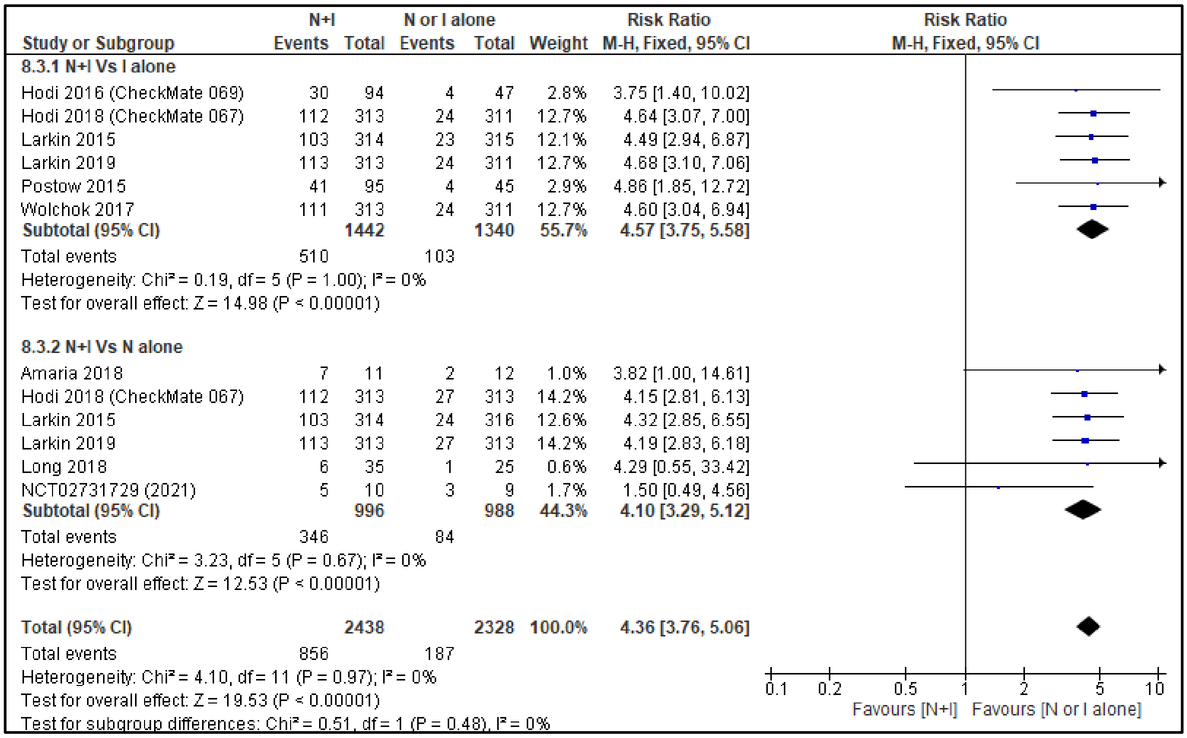

Figure S6: Forest plot for liver adverse effects.

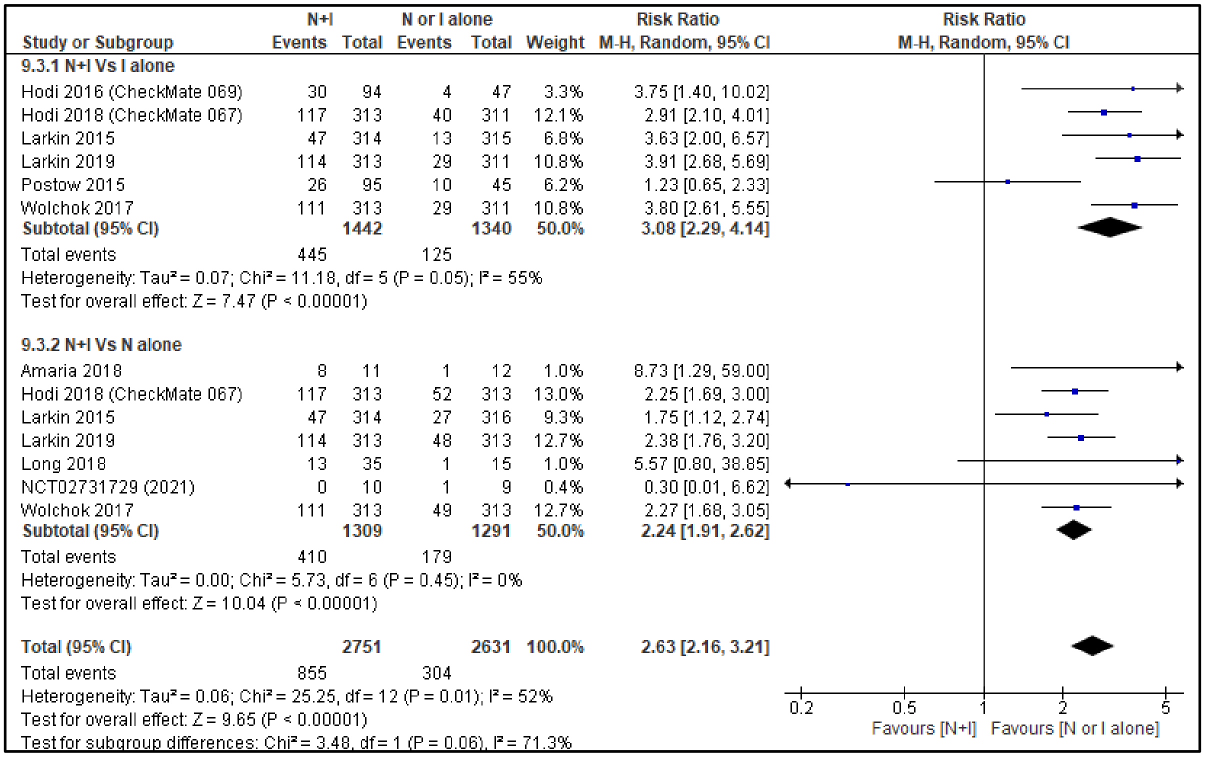

Figure S7: Forest plot for endocrine adverse effect
